# Highly Stable PLA/Gelatin Bionanocomposites for Enhanced Delivery of Chloroaluminum Phthalocyanine for Photodynamic Therapy against SK-MEL-28 Cells

**DOI:** 10.1021/acsomega.6c05499

**Published:** 2026-10-13

**Authors:** Janicy A. Carvalho, Jéssica A. R. Ambrósio, Milton Beltrame Junior, Vitor L. M. Marmo, Leandro J. Raniero, Lucas F. Lyra, Kumiko K. Sakane, Juliana G. Pinto, Tassia J. Martins, Juliana Ferreira-Strixino, Andreza R. Simioni

**Affiliations:** 67655 Universidade do Vale do Paraíba, São José dos Campos, São Paulo 12244-000, Brazil

## Abstract

This study reports the development of a biodegradable poly­(lactic acid) and gelatin (nPLA-G) bionanocomposite as a drug delivery system for chloroaluminum phthalocyanine (ClAlPc) in photodynamic therapy (PDT). Using a hybrid desolvation/emulsification technique, we engineered spherical nanoparticles with an average diameter of 753.3 nm and a positive zeta potential of +8.90 mV. ClAlPc-loaded nanoparticles (nPLA-G/ClAlPc) were prepared using the same process, with a mean diameter of 809.3 nm and a zeta potential of +16.10 mV. The system achieved an exceptional encapsulation efficiency of 90.89% and maintained colloidal stability for over 270 days. In vitro assays showed a pH-dependent release profile, optimizing drug availability in tumoral microenvironments. The nPLA-G/ClAlPc system showed no dark toxicity but reached 97% cytotoxicity in SK-MEL-28 melanoma cells upon light activation (1.0 μmol L^–1^). These preliminary results demonstrate that the bionanocomposite effectively overcomes ClAlPc hydrophobicity, serving as a promising drug delivery system with preliminary photodynamic efficacy *in vitro*.

## Graphical abstract

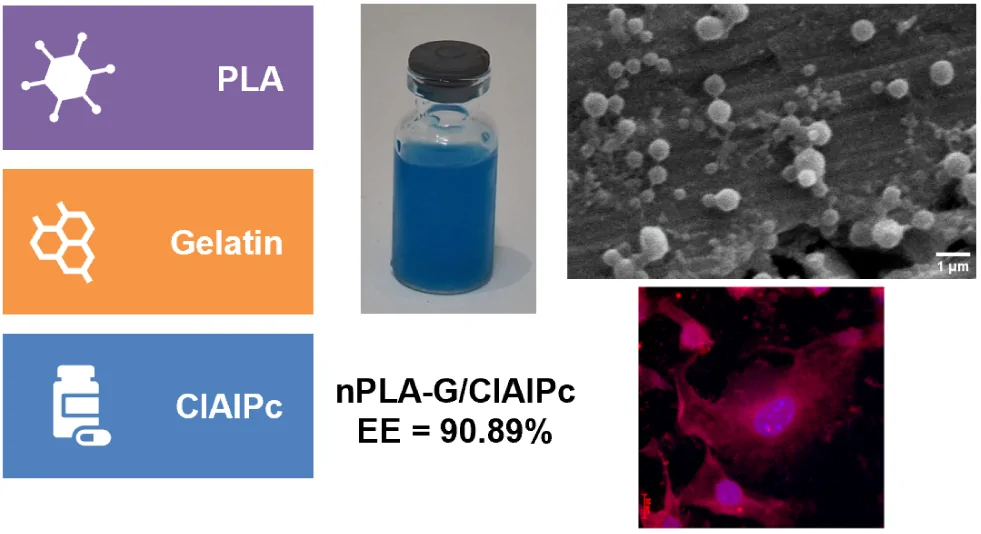

## Introduction

Cancer remains a global health challenge where, despite the success of standard treatments, issues like metastasis, relapse, and cellular resistance persist.^1–3^ In this context, PDT has emerged as a minimally invasive modality for cancer treatment that utilizes a photosensitizer (PS), light, and oxygen to generate reactive oxygen species (ROS), inducing localized cell death. Initially, a nontoxic PS is administered topically or injected systemically. The PS responds to specific wavelengths of light, releasing its excited-state energy to the surrounding oxygen. These actions produce ROS, particularly reactive singlet oxygen, which can destroy tumor cells.^3–7^

Among second-generation PSs, phthalocyanines, such as chloroaluminum phthalocyanine (ClAlPc), stand out due to their high oxidative power, high efficiency in singlet oxygen production, chemical stability that provides enhanced cell penetration and intense absorbance within the therapeutic window (600–800 nm).^8–10^ However, phthalocyanines are hydrophobic compounds and require a drug delivery system (DDS) to overcome these challenges.^11^ A DDS is a formulation that enables the delivery of active chemicals into the body by controlling the drug’s dose, timing, and release, thereby improving its efficacy and safety.^12,13^ Among DDS types, polymer-based nanoparticles exhibit distinct features and mechanisms that modify the drug’s physicochemical properties, thereby affecting its biopharmaceutical and pharmacokinetic behavior.^14,15^ Besides, polymeric nanoparticles offer versatility in terms of surface modification, enabling targeted delivery and controlled release.^16^

Poly­(lactic acid) (PLA) is a synthetic polymer approved by the Food and Drug Administration (FDA) for human use. It is employed in pharmaceutical and medicinal applications, such as DDS, stents, dialysis media, and surgical sutures, as it biodegrades into nontoxic compounds.^14,17^ Due to their potential to alter biodistribution, PLA nanoparticles have been extensively studied for the administration of both hydrophilic and hydrophobic active medicinal ingredients.^18^ On the other hand, gelatin is a naturally occurring macromolecule derived from the hydrolysis of collagen; it is nontoxic, biodegradable, and excellent for nanoparticle preparation.^19,20^ Scaffolds based on PLA/gelatin have been widely employed as matrices for DDS or tissue engineering;^21^ however, to the best of our knowledge, this work represents a novel development of hybrid PLA/gelatin nanoparticles as a DDS for a phthalocyanine PS in PDT directed at melanoma cells.

Therefore, this work aimed to design a PLA and gelatin nanoparticle (nPLA-G) as a DDS and incorporate ClAlPc (nPLA-G/ClAlPc) for PDT applications. The formulations underwent thorough physicochemical characterization and primary in vitro photodynamic evaluation in the SK-MEL-28 cell line as an alternative cancer treatment option.

## Materials and Methods

### Materials

Poly­(D,L-lactic acid) (PLA; average Mn 40,000), Gelatin (Porcine Skin type A Bloom 300), ClAlPc, trypan blue, and Dulbecco’s modified Eagle medium (DMEM) were purchased from Sigma-Aldrich (Brazil). ProLong Diamond Antifade Mountant with DAPI (40,6-diamidino-2-phenylindole, dihydrochloride), fetal bovine serum (FBS) and Penicillin/Streptomycin solution were purchased from Thermo Fisher Scientific Inc. (USA). Organic solvents were purchased from Synth (Brazil).

### Synthesis of PLA-Gelatin Nanoparticle (Bionanocomposite)

The nPLA-G was prepared by combining the desolvation^22^ and emulsification technique.^23^ First, the gelatin went through the desolvation process. For that, 25 mL of acetone was rapidly added to 25 mL of gelatin aqueous solution (0.02 g·mL^–1^), heated to 50 °C. After 2 min, the supernatant was discarded, and the precipitated gel was redissolved in 25 mL of water at 50 °C, and the pH was adjusted to 3.0. Then, this solution was emulsified with 10 mL of a chloroform solution containing PLA (0.03 g·mL^–1^) at high speed with an ultraturrax setup. Immediately after, 0.1 mL of a glutaraldehyde aqueous solution (25%) was added. The emulsification formed was gently stirred at room temperature until the organic solvent was removed. The nanoparticles were purified by applying three cycles of centrifugation at 6500 rpm (CF16RN, High-speed Micro Centrifuge, HITACHI, Japan). For the nPLA-G/ClAlPc, the same method was used. However, a ClAlPc solution in ethanol/DMSO (90:10 v/v) (1 mg·mL^–1^) was added at the organic phase before the emulsification. Figure 1 shows a schematic representation of the bionanocomposite’s preparation.

**Figure 1:**
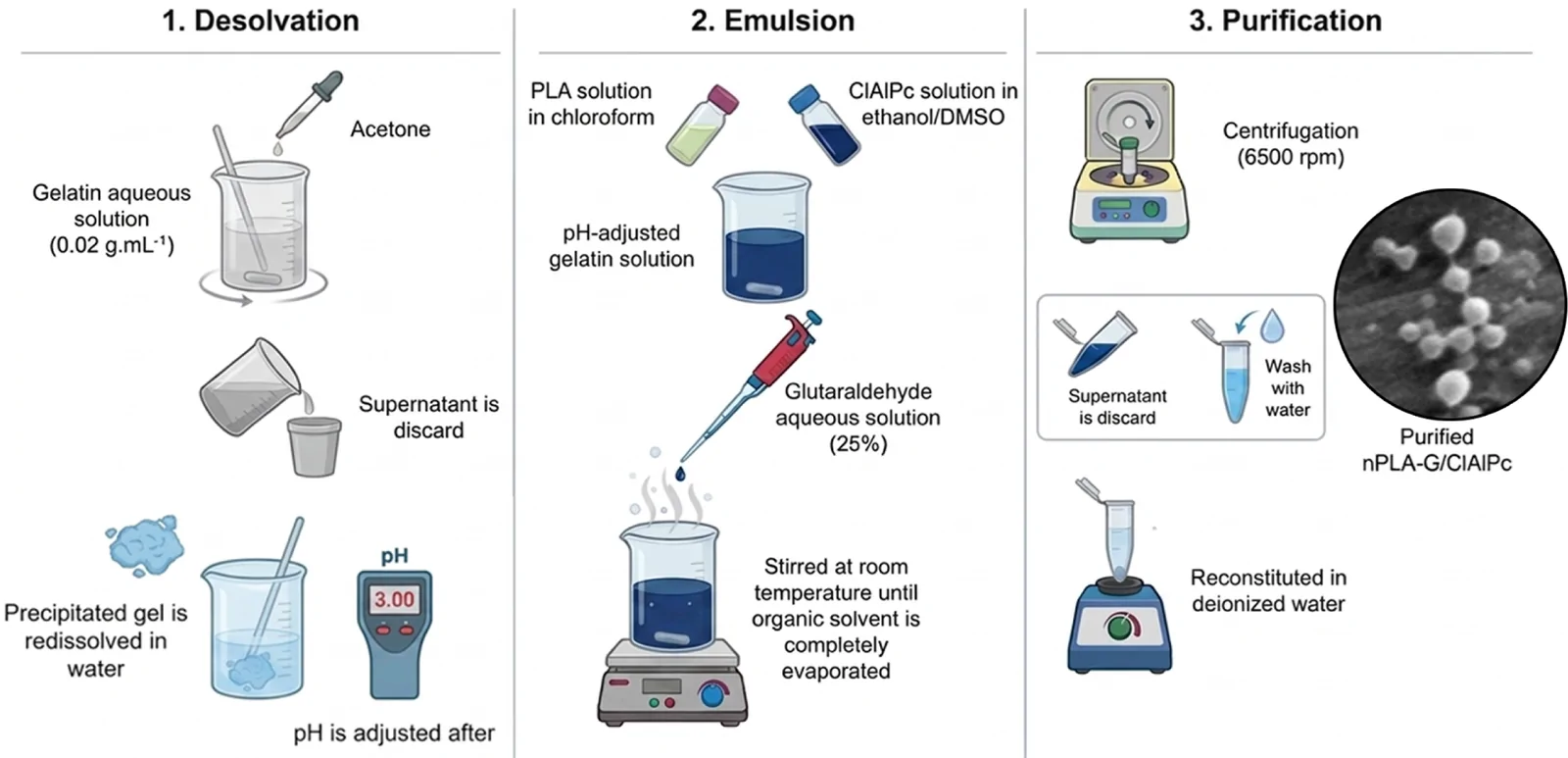
Schematic representation of the bionanocomposite’s preparation.

### Morphological Characterization

The external morphology of nPLA-G and nPLA-G/ClAlPc was performed by scanning electron microscopy (SEM) using the EVO-MA10 equipment (Zeiss, Germany). The samples were coated with a layer of gold and analyzed at an acceleration voltage of 10 kV.

### Energy Dispersive Spectroscopy

Energy-dispersive spectroscopy (EDS) was performed using the EVO-MA10 (Zeiss, Germany). This technique enables qualitative analysis of the chemical elements present in the evaluated sample.

### Physicochemical Characterization

Dynamic light scattering (DLS) was used to characterize the size distribution and zeta potential of the nanoparticles. The Zetasizer Nano ZS (Malvern, UK) equipment was used for the study. The particles were dispersed in distilled water for DLS measurements, and measurements were taken at a scattering angle of 90° at room temperature. For the stability test, the samples’ size and zeta potential were measured at distinct storage times for 270 days.

### Fourier Transform Infrared Spectroscopy

Infrared absorption spectra were obtained in the range 4000–450 cm^–1^ with a resolution of 4 cm^–1^ in transmission mode at a controlled temperature of 20 °C. The spectrophotometer used was a Spectrum Two Fourier Transform Infrared (FTIR) Spectrometer with attenuated total reflectance (ATR) technology (PerkinElmer, USA).

### Raman Spectroscopy

Raman spectroscopy also allowed the identification of a material’s chemical structure. The equipment used was an RFS 100/S (Bruker Corporation, USA), and the readings were taken over the range 50–3500 cm^–1^ using a 1064 nm laser.

### X-ray Diffraction

X-ray diffraction (XRD) made it possible to identify the phases in the samples and their crystalline structure. The XRD spectra were recorded using an XRD-6000 diffractometer (Shimadzu Instruments, Japan) with a Ni-filtered Cu Kα radiation source and a scan speed of 1°/min^–1^. A voltage of 40.0 kV, a current of 30.0 mA, and a wavelength of 1.54 Å were employed.

### Thermogravimetric Analysis

Thermogravimetric analysis (TGA) enabled assessment of mass loss as a function of the sample’s temperature. The thermal characteristics of gelatin, PLA, and nPLA-G were evaluated utilizing an STA 449 F3 Jupiter (NETZSCH, Germany) analyzer with a heating rate of 10 K·min^–1^ across a temperature range of 25 to 1000 °C.

### Steady-State Spectroscopy

The UV–vis studies enabled comparison of ClAlPc spectra (free and encapsulated in the system), allowing evaluation of changes due to encapsulation. Following the development of the quantification standard curve, the percentage of assets incorporated in the broadcasting system was quantified. Beer’s law was followed throughout the spectrophotometric analyses. The data were analyzed using least-squares regression. The spectrophotometer was a Cary 50 BIO-Varian (Inc. Scientific Instruments, USA).

Fluorescence analysis was used for the same purpose, and the VANTAstar microplate reader (BMG LABTECH, Germany) was utilized. The fluorescence experiments used an excitation wavelength of 616 nm, and fluorescence emission spectra were recorded between 650 and 740 nm.

### Determination of Encapsulation Efficiency

The encapsulation efficiency (EE) of ClAlPc was estimated using UV–vis spectroscopy.^24^ In brief, 1 mL of 0.1 mg·mL^–1^ of nPLA-G/ClAlPc was dissolved in DMSO (1:1, v/v) and sonicated for 5 min to release all ClAlPc. The samples were centrifuged at 6500 rpm (CF16RN, High-speed Micro Centrifuge, HITACHI, Japan), the supernatant was collected, and the ClAlPc concentration was determined using spectroscopy techniques. The absorbance measurements were performed using a Cary 50 BIO-Varian (Inc. Scientific Instruments, USA). The encapsulation efficiency was found using the following eq 1:

1 EE=(M1/M2)×100

where EE is the encapsulation efficiency, M1 is the mass of ClAlPc in the nanoparticles quantified by UV–visible spectroscopy, and M2 is the mass of ClAlPc weighed and placed in the formulation’s preparation.

The absorbance of the sample solutions was measured at 676 nm, the maximum absorption wavelength (λmax) of ClAlPc.

### Evaluation of the In Vitro Release Studies

The in vitro release studies were examined under two different conditions (pH 7.4 and pH 5.0), which simulated the pH of normal and cancer cells, respectively.^25^ Briefly, 1 mL of nPLA-G/ClAlPc was dispersed in 2 mL of phosphate-buffered saline (PBS) at pH 5.0 and 7.4, respectively, at 37 °C, and placed on a shaker. At predefined intervals, the suspension was centrifuged at 10000 rpm (CF16RN, High-speed Micro Centrifuge, HITACHI, Japan) for 5 min, and 2 mL of the supernatant was taken. A new buffer was added to maintain a constant volume. The amount of released ClAlPc was determined by measuring absorbance and calculating it using the calibration curve and eq 2.

2 Drugrelease(%w)=(RPS/LPS)×100

where RPS is the amount of released ClAlPc and LPS is the loaded ClAlPc.

### Cell Culture

The cell line SK-MEL-28 (ATCC #HTB-72) is melanocytes isolated from the skin tissue and has applications in cancer and toxicology research. The SK-MEL-28 cells were seeded in DMEM supplemented with 10% FBS and 1% Penicillin/Streptomycin solution. The incubator was set to 37 °C with 5% CO_2_ and a humid environment. The medium was changed on alternate days according to the cells’ metabolic needs.

### Internalization of the nPLA-G/ClAlPc

For the internalization tests, the cells were removed from the culture flask with a cell scraper and adhered to circular coverslips placed at the bottom of 24-well plate wells at a concentration of 1 × 10^5^ cells. They were maintained overnight in an incubator at 37 °C and 5% CO_2_. Then, the medium was removed, and the ClAlPc or the nPLA-G/ClAlPc was added at concentrations of 0.35, 0.70, and 1.0 μmol L^–1^, and maintained at 37 °C. After 1 h, the samples were washed twice with PBS and fixed for 15 min with 4% paraformaldehyde dilution in PBS. The coverslips were then removed, mounted with ProLong containing DAPI, and examined under an LSM 700 (Zeiss, Germany) confocal microscope. For the ClAlPc and nPLA-G/ClAlPc the excitation was used at 555 nm, and for the DAPI marker, at 405 nm.

### Cell Viability Analysis by the Trypan Blue Exclusion Method

The Trypan blue exclusion method, which relies on the principle that viable cells exclude the dye while dead cells remain stained, was used to assess cell viability.

To evaluate the nPLA-G/ClAlPc and free ClAlPc, SK-MEL-28 was seeded at 1 × 10^5^ cells/well in 96-well plates. PBS was used to dilute the formulations to 0.35, 0.70, and 1.0 μmol L^–1^. After 1 h of incubation at 37 °C and 5% CO_2_, the formulations were removed and replaced with fresh PBS. Then, the dark groups were kept in the dark, and the irradiated groups received a dose of 10 J·cm^–2^. For irradiation, the BioPDI Irrad-Led (BioPDI, Brazil) biotable equipment consisting of 54 LEDs was used, with an irradiance of 36.8 mW at 660 nm. Immediately after irradiation, the PBS was completely removed, fresh DMEM supplemented with 10% FBS (free of formulations) was added, and the plates were maintained at 37 °C with 5% CO_2_ for 24 h.

After 24 h, the medium was removed, 0.2% Trypan Blue was added, and the mixture was incubated for 5 min. The Trypan was then removed, PBS was added, and images of the cells were captured in 10 random fields using the Zeiss Axio Vert inverted microscope A1 (Zeiss, Germany). As controls, we created a set of wells with SK-MEL-28 and nPLA-G at the same ratio as the most concentrated test (nPLA-G/ClAlPc), and a set with only SK-MEL-28, without any formulations. The cell count was performed with ImageJ, and the percentage of live cells was calculated using eq 3.

3 Viablecells(%)=(Totallivingcells/Totalcountcells)×100

### Statistical Analysis

Three separate experiments were conducted for each parameter under analysis, and the results are presented as the mean ± SD. For statistical analysis, one-way analysis of variance (ANOVA) was employed. To investigate statistical significance (*p* < 0.05), Tukey’s post hoc adjustment for multiple comparisons was used. Statistical analyses were performed using GraphPad Prism 7 software (GraphPad Software, USA).

## Results

This study involved preparing PLA/gelatin nanoparticles for ClAlPc encapsulation using a combination of desolvation and emulsification techniques. The nPLA-G shown in the aqueous solution in Figure 2a is the result of this technique. The PS, a hydrophobic compound, was added in the organic phase with PLA to obtain the nPLA-G/ClAlPc (Figure 2b). The morphology of the nPLA-G (Figure 2c) and nPLA-G/ClAlPc (Figure 2d) were examined by SEM. The EDS spectrum in Figure 2f was recorded for the area marked with a purple rectangle in Figure 2e, indicating the signals of C and O elements for the nPLA-G. For the nPLA-G/ClAlPc (Figure 2h), the EDS spectrum was recorded for the area marked in Figure 2g, and the signals of C, O, Al, and Cl elements were found.

**Figure 2:**
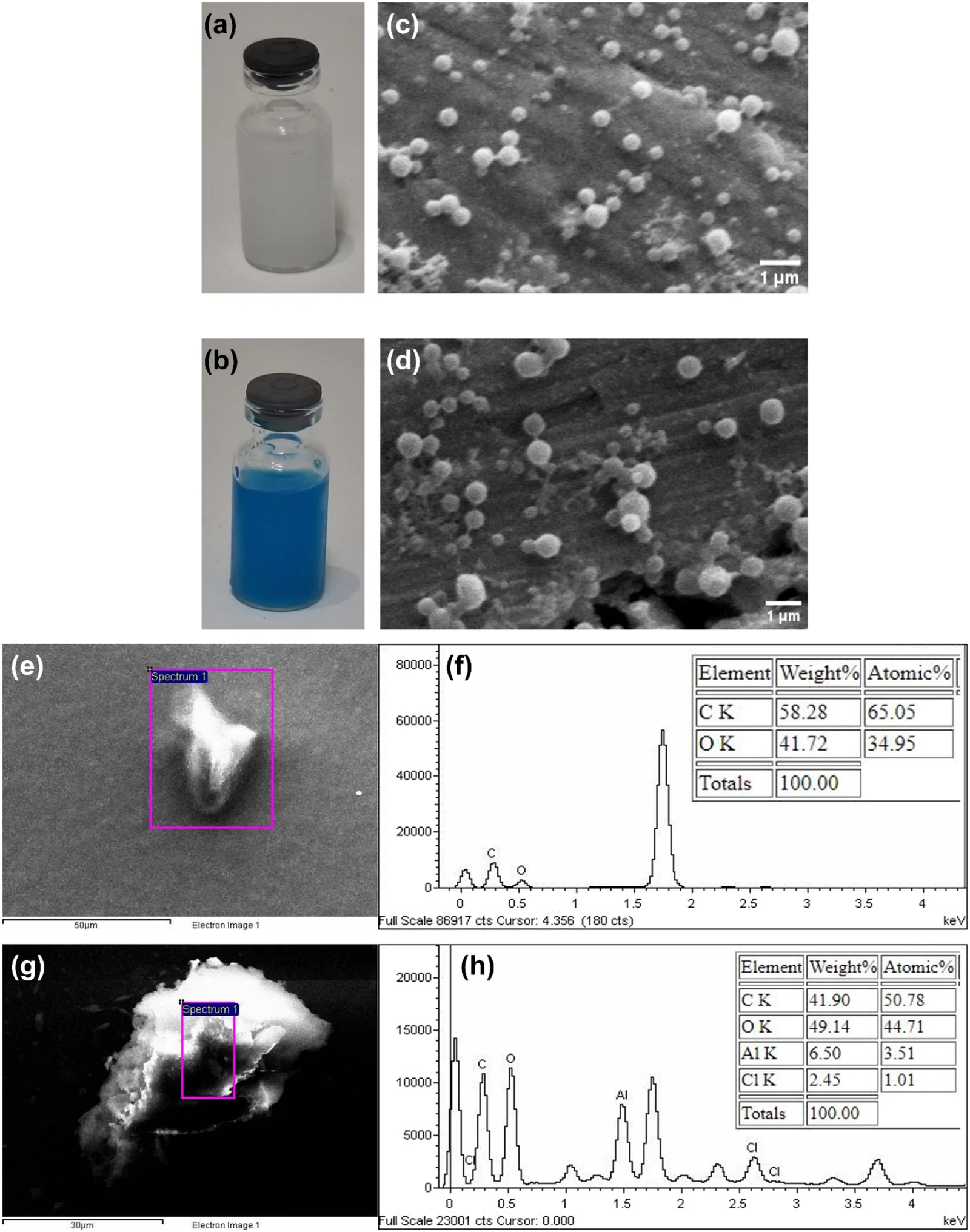
(a) nPLA-G; (b) nPLA-G/ClAlPc; (c) nPLA-G SEM micrography; (d) nPLA-G/ClAlPc SEM micrography; (e) nPLA-G with the area analyzed marked; (f) nPLA-G EDS spectrum; (g) nPLA-G/ClAlPc with the area analyzed also marked; (h) nPLA-G/ClAlPc EDS spectrum.

The nPLA-G exhibited an average diameter of 753.3 ± 224.8 nm (PdI = 0.152) (Figure 3a) and a zeta potential of +8.90 ± 2.99 mV. For the nPLA-G/ClAlPc, the mean diameter increased to 809.3 ± 192.7 nm (PdI = 0.047) (Figure 3b) and zeta potential of +16.10 ± 3.24 mV. For the stability test, the samples’ size distribution and zeta potential were collected for 270 days. Figure 3c and Figure 3d show the results for the nPLA-G and nPLA-G/ClAlPc, respectively. Furthermore, a statistical analysis (one-way ANOVA) was conducted comparing the hydrodynamic diameters over 270 days.

**Figure 3:**
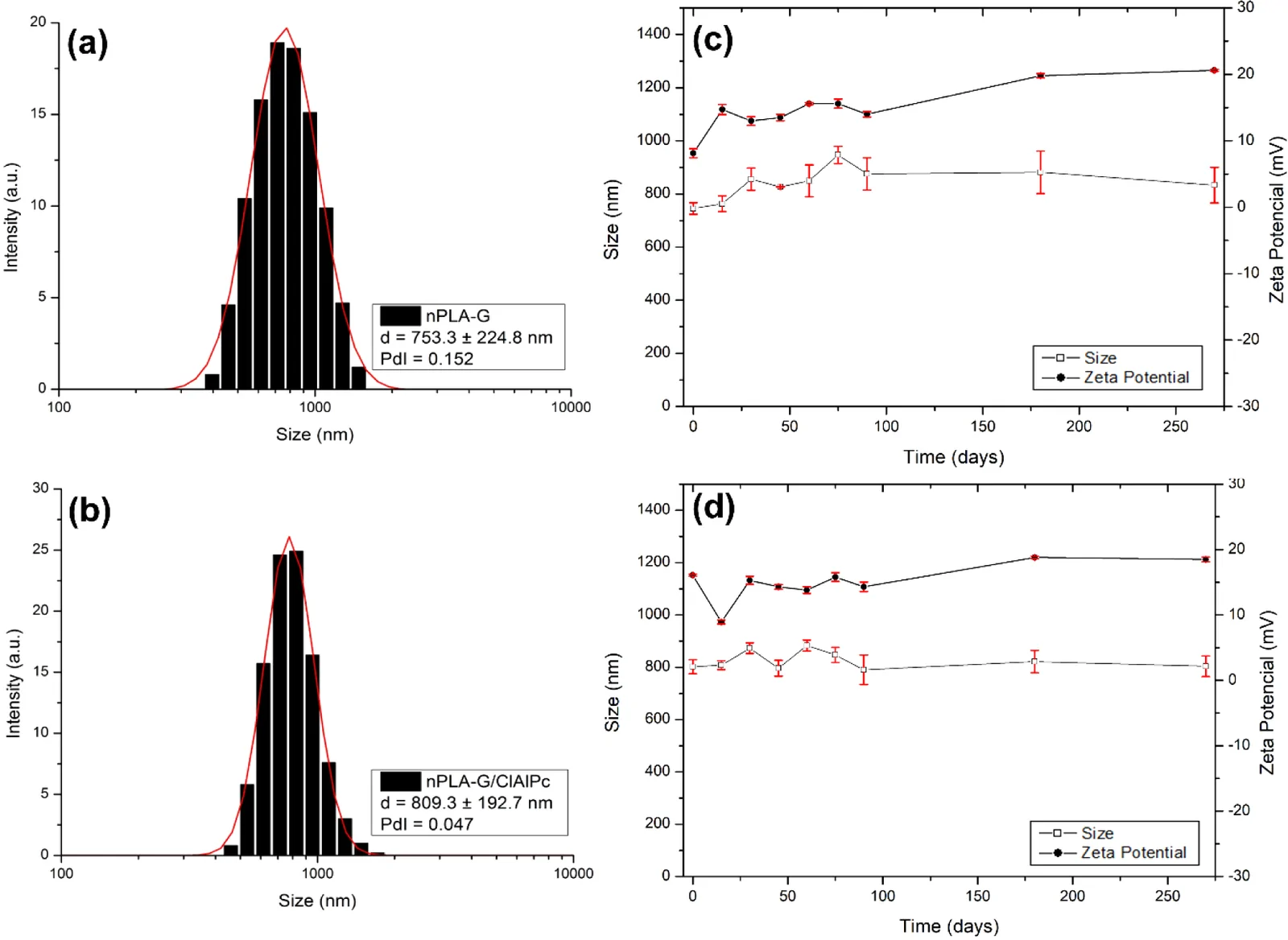
(a) nPLA-G and (b) nPLA-G/ClAlPc size distributions; (c) nPLA-G and (d) nPLA-G/ClAlPc hydrodynamic size and zeta potential monitored for stability during 270 days. For the stability test, data are expressed as mean ± SD (*n* = 3).

FTIR analysis was performed on nPLA-G compared to the individual components (PLA and gelatin) (Figure 4a). Figure 4b compares the nPLA-G and ClAlPc spectra with the nPLA-G/ClAlPc to verify the encapsulation. Raman spectroscopy was also used to analyze nPLA-G and to compare it with PLA and gelatin (Figure 4c).

**Figure 4:**
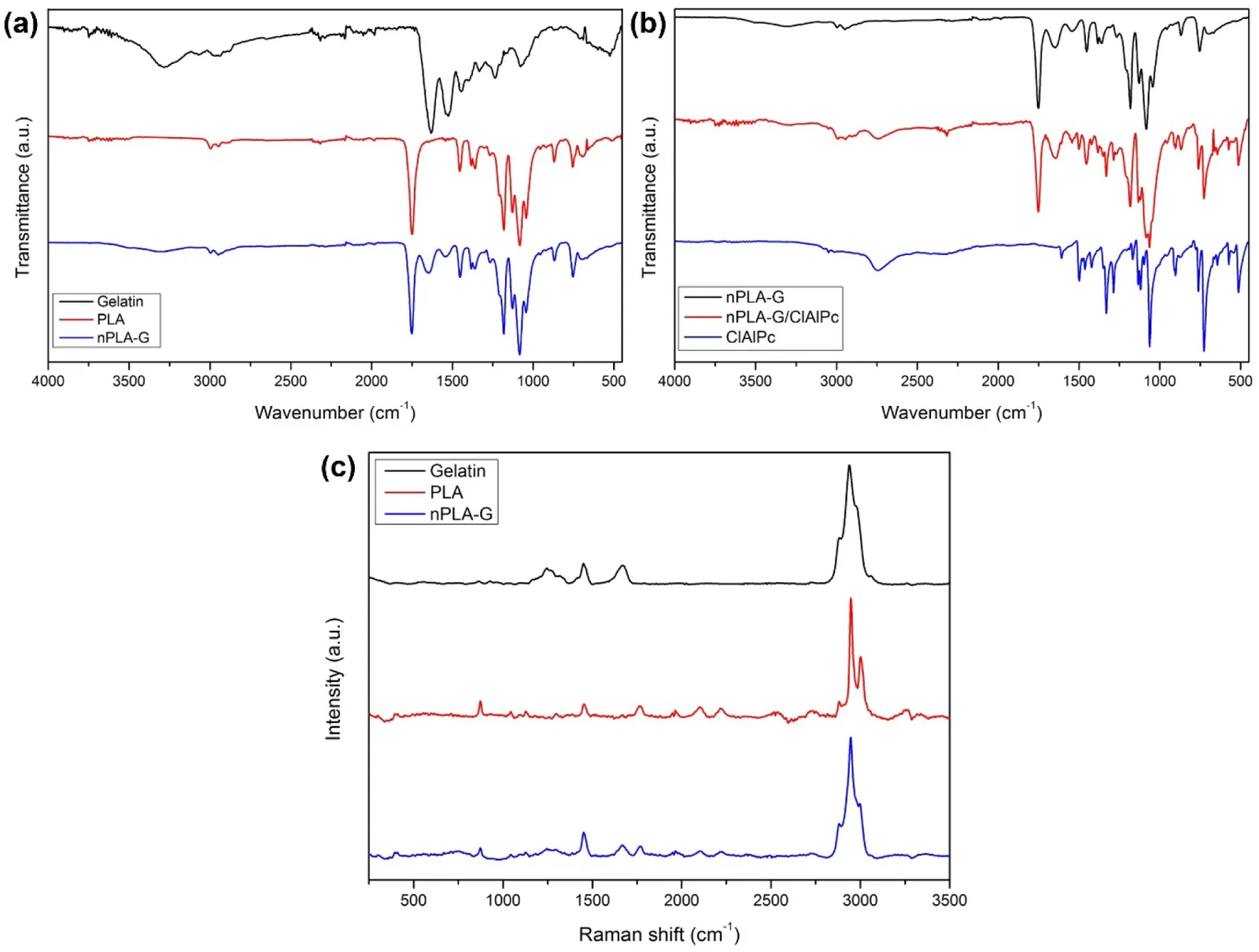
(a) FTIR spectra for the nPLA-G and its components; (b) FTIR spectra for the nPLA-G/ClAlPc in comparison with the nPLA-G and ClAlPc; (c) Raman spectroscopy for the nPLA-G and its components.

The XRD pattern of the PLA, gelatin, and nPLA-G are shown in Figure 5a. PLA’s XRD pattern revealed two distinctive major peaks at 16.8° and 19.2°. Gelatin appeared as taro peaks around 20°. The nPLA-G exhibited peaks at 16.8° and 19.2°. In Figure 5b, we compared the XRD patterns of nPLA-G and ClAlPc with that of nPLA-G/ClAlPc to verify the encapsulation effect on the crystalline structure. The X-ray diffraction diagram of ClAlPc powder showed a firm diffraction peak at 26°.

**Figure 5:**
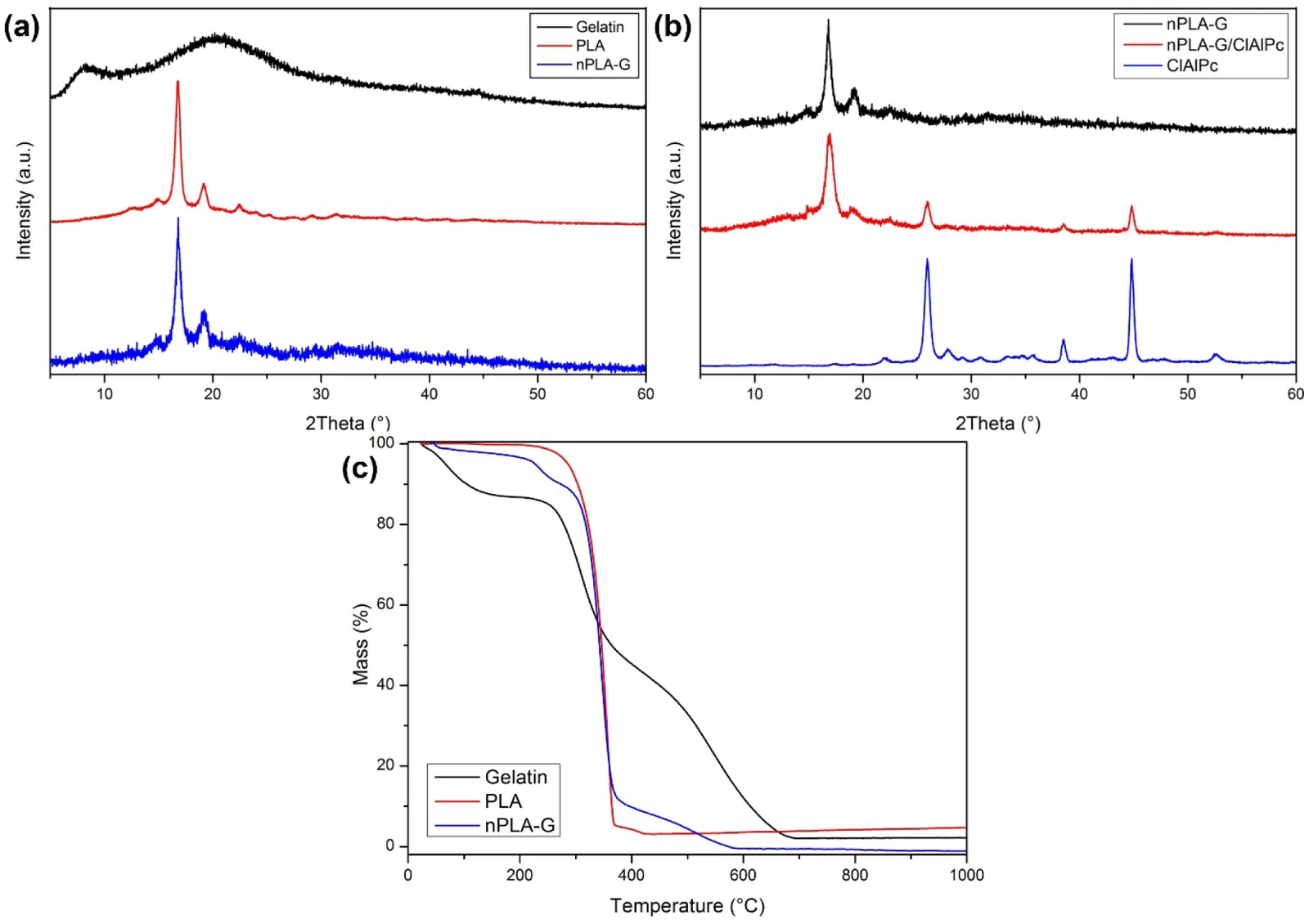
(a) XRD pattern of the PLA, gelatin, and nPLA-G; (b) Comparison between the nPLA-G and ClAlPc XRD patterns with the nPLA-G/ClAlPc; (c) TGA analysis of gelatin, PLA and nPLA-G.

TGA of gelatin, PLA, and nPLA-G are shown in Figure 5c. The TGA profile of PLA has only one step at 366.4 °C. The gelatin, on the other hand, has three steps: around 100 °C, 303.6 °C, and 556.5 °C. The nPLA-G exhibited a three-stage degradation profile: 223.5 °C, 323.3 °C, and around 500–600 °C.

The absorption spectra of ClAlPc (2 μmol L^–1^) standard and extracted from nPLA-G/ClAlPc in DMSO are illustrated in Figure 6a. UV–vis spectroscopy measured the concentration of PS on the bionanocomposites by doing a calibration curve (Figure 6b–6c). The spectrophotometric technique showed satisfactory linearity in the concentration range (0.5–3.5 μmol L^–1^) with a high correlation coefficient (R^2^ = 0.99864). The straight-line Equation was absorbance = 0.23871 × [ClAlPc, concentration in μmol L^–1^] + 0.01274. Figure 6d reports the fluorescence emission spectrum of both ClAlPc-free and nPLA-G/ClAlPc. We also determined the encapsulation efficiency of the suggested system and had a result of 90.89 ± 7.55%.

**Figure 6:**
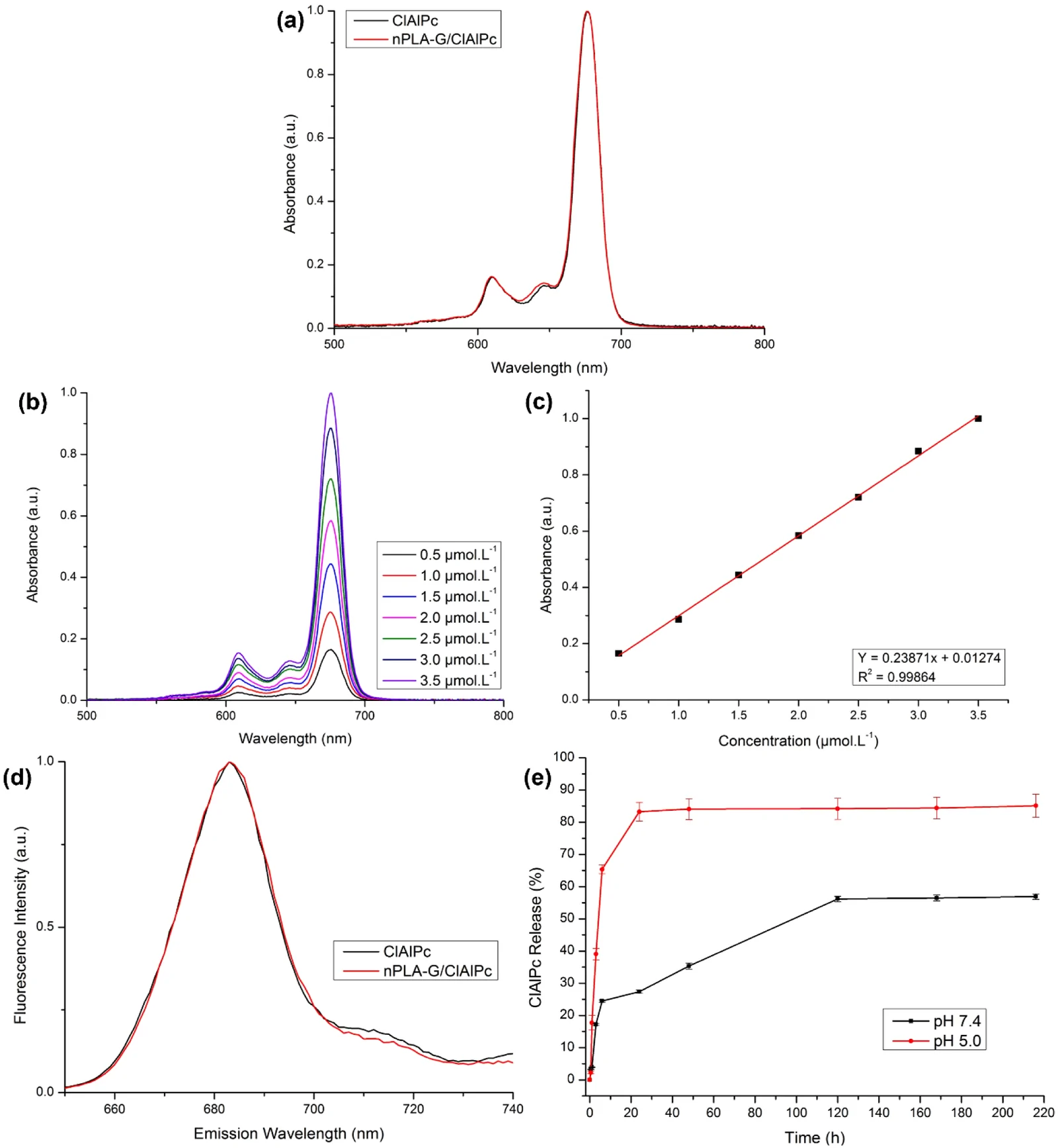
(a) UV spectra of ClAlPc in DMSO 10% and nPLA-G/ClAlPc; (b) Absorption spectra of ClAlPc at different concentrations (0.5–3.5 μmol L^–1^); (c) Plot of ClAlPc concentration vs absorbance; (d) Fluorescence spectra of ClAlPc and nPLA-G/ClAlPc; (e) In vitro drug release studies of nPLA-G/ClAlPc in different pHs.

Before proceeding with the drug release analysis, it is essential to understand that the ClAlPc free is insoluble in water and PBS.^26^ Therefore, we carried out the study only for the nPLA-G/ClAlPc, and the obtained drug release behavior is in Figure 6e.

Confocal microscopy was used to observe the internalization of nPLA-G/ClAlPc and the free ClAlPc for comparison (Figure 7a). For the cell viability study, the same samples and concentrations were tested with the trypan blue exclusion method (Figure 7b). To discard any intrinsic toxicity or phototoxicity from the isolated components of the therapy, critical control groups were evaluated under identical conditions.

**Figure 7:**
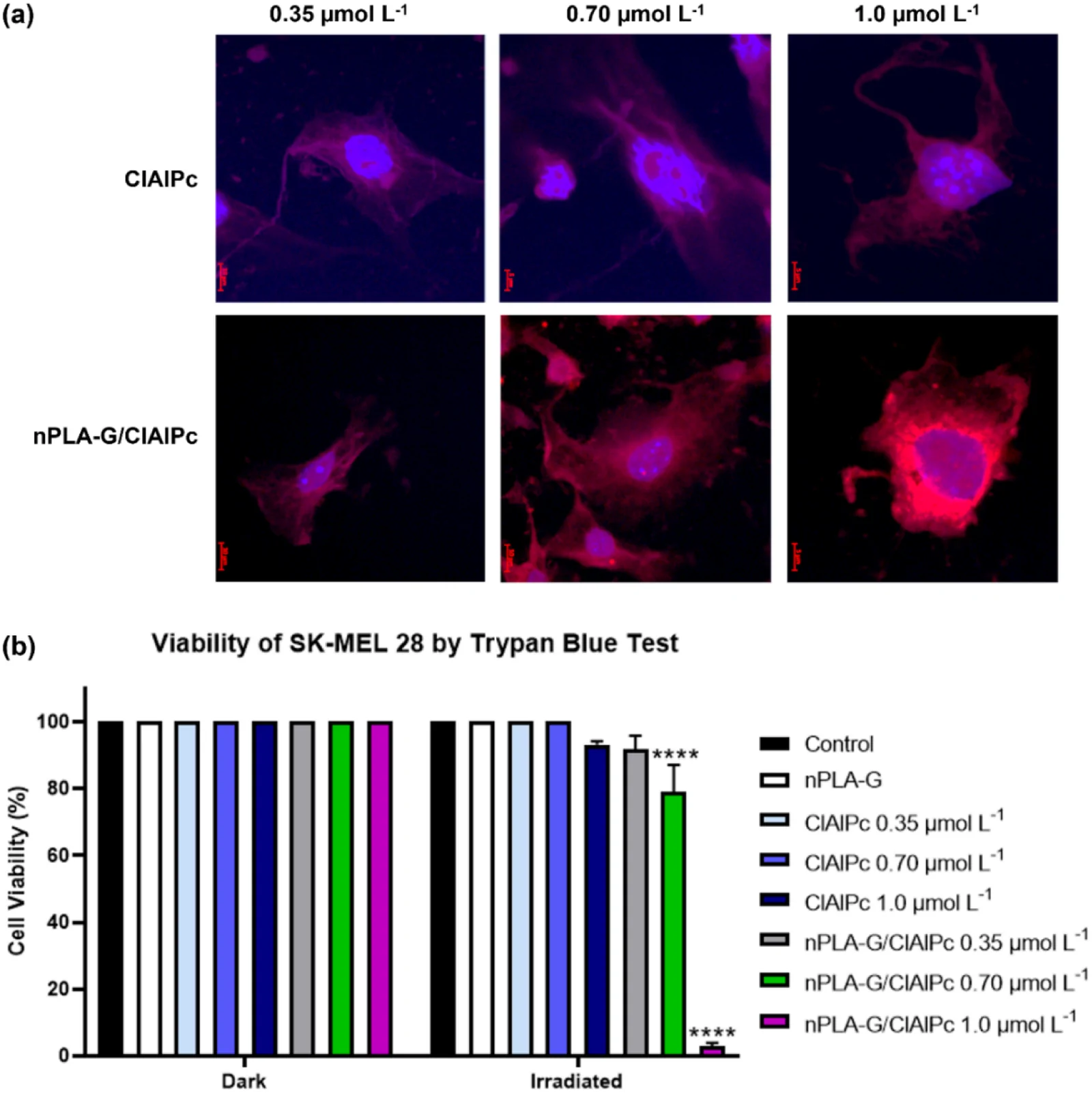
(a) Confocal microscopy showing blue fluorescence from the DAPI attached to the cell’s DNA and red fluorescence from the excited ClAlPc or the nPLA-G/ClAlPc; (b) Evaluation of SK-MEL-28 cell viability. The experimental design includes essential control groups to evaluate isolated effects: Control (Irradiated) represents cells exposed to light alone without nanoparticles to evaluate potential light-induced toxicity; nPLA-G (Irradiated) represents cells treated with empty nanoparticles under light exposure to rule out nanocarrier phototoxicity. Both controls were also evaluated without irradiation (Dark). Data are expressed as mean ± SD (*n* = 3). There is a significant difference (*p* < 0.0001) between the irradiated control sample and the nPLA-G/ClAlPc in the concentrations of 0.70 and 1.0 μmol L^–1^ (****).

## Discussion

Techniques for preparing polymeric nanoparticles are significant in biomedical research, particularly for drug delivery applications.^27^ In this study, nanoparticles were engineered by combining desolvation and emulsification. Gelatin desolvation traditionally involves a two-step process to regulate protein unfolding and suppress aggregation by balancing repulsive and attractive forces.^28,29^ In this study, we used only the first step of gelatin desolvation to separate the high-molecular-weight fraction, reducing and standardizing its size for the next stage, in which the emulsification technique was performed to form the gelatin-PLA bionanocomposite.

Emulsification is the first and most widely used technique for creating polymeric nanoparticles. The polymer solution is emulsified into an aqueous phase in the first step. The second step is solvent evaporation, which leads to polymer precipitation and nanoparticle formation. When the emulsion is complete, the solvent is continuously evaporated, the nanoparticles are separated by ultracentrifugation, and any remaining residue is thoroughly washed with water. Changing the dispersion agent’s kind and concentration, the aqueous and organic phases’ viscosity and the stirring speed can all affect the diameter of the nanoparticles.^30^ In this work, we used a single-emulsion, i.e., oil-in-water (o/w), in which the organic phase was a PLA solution in chloroform, and the aqueous phase was the previously desolvated gelatin solution.

The nanoparticles developed in this study were spherical with smooth surfaces, and no aggregation was observed. The addition of PS did not affect the morphological properties of the nanoparticles.

In the EDS, the signals of C and O elements were observed for the nPLA-G. Gelatin is obtained when collagen is partially hydrolyzed by heat, acid, or alkaline. As a result, it shares a nearly identical basic structure with collagen. Generally, like collagen, gelatin is characterized by the repetition of the triplet (Glycine-X-Y)­n, where X and Y are usually proline and hydroxyproline, respectively.^31^ Conversely, PLA is derived from lactic acid; it is recognized as an aliphatic polyester and is commonly produced from hydroxyl acids.^32^ Thus, both are composed of carbon, hydrogen, oxygen, and nitrogen (in the case of gelatin), which explains the observed signals. Meanwhile, for the nPLA-G/ClAlPc, the signals of C, O, Al, and Cl elements were found. ClAlPc is a nonplanar molecule with a central group outside the molecular π-plane and a molecular formula of ClAlC_32_H_16_N_8_.^33^ The signals of Al and Cl in the nPLA-G/ClAlPc confirm the encapsulation of the PS.

Polymer nanoparticles range from 1 to 1000 nm.^34^ In this study, nPLA-G and nPLA-G/ClAlPc exhibited low PDI and nanoscale size. A low PDI indicates low particle size disparity. A drug’s effectiveness may be undermined by size nonuniformity, which results in inefficient particle circulation, distribution, and clearance.^35^ By employing the double emulsification process, Wongrakpanich et al.^36^ synthesized particles with a median size of 3.12–7.12 μm using PLA and poly­(lactide-*co*-glycolide). While conventional DDS often prioritizes sub-200 nm dimensions to evade rapid systemic clearance, larger submicron carriers offer distinct advantages for localized and regional oncological therapies. From a materials perspective, keeping the hybrid biopolymer architecture within the submicron scale balances the fast nucleation rates, preventing irreversible aggregation. From a physiological standpoint, this size profile is optimized for localized retention and sustained topical or intratumoral PDT efficacy rather than systemic circulation.^37^ Given the average particle size of approximately 800 nm, systemic intravenous delivery is not the intended route for this formulation. Instead, this bionanocomposite size profile is particularly tailored for localized, topical, or intratumoral administration, ensuring enhanced local retention and preventing rapid systemic dispersion. Furthermore, as demonstrated by Chesneau et al.,^37^ particles within the 200 to 1200 nm range are successfully internalized by tumor cells, making it appropriate for the proposed application.

The nanoparticles’ surface zeta potential was positive. The zeta potential represents the particle’s surface charge, which is affected by modifications in the interface with the dispersing medium, the solvation effect, the dissociation of functional groups on the particle’s surface, or the adsorption of ionic species in the aqueous dispersion medium.^33^ PLA is polyester, and because of that, PLA nanoparticles generally have a negative zeta potential due to carbonyl and carboxyl terminals.^38,39^ However, the presence of amino groups often confers a positive zeta potential on gelatin nanoparticles.^40^ We can conclude that the surfaces of nPLA-G and nPLA-G/ClAlPc have more gelatin chemical groups that contribute to the zeta potential.

Surface zeta potential is a key physicochemical factor influencing the long-term physical stability of colloidal suspensions. Traditionally, a magnitude exceeding ±30 mV is cited as a benchmark for adequate colloidal stability to prevent aggregation caused by occasional collisions from Brownian motion.^33^ Crucially, however, this empirical rule applies strictly to lyophobic colloidal systems stabilized purely by electrostatic repulsion, as governed by classic DLVO theory. In composite systems incorporating macromolecular polymers, stability is governed by electrosteric mechanisms, where steric hindrance plays a primary or dominant role, making surface charge alone an inadequate predictor of colloidal fate.^41^

The two most common techniques for stabilizing nanoparticles are electrostatic stabilization and steric stabilization. Steric stabilization, often known as polymer stabilization, is a common approach for stabilizing colloidal dispersions. This method is based on steric hindrance generated by organic molecules on the particle’s surface, forming a protective coating that prevents particle aggregation. Gelatin is a well-known stabilizer used in suspensions and emulsions and can accomplish stability by acting as a steric barrier.^42,43^ During the hybrid desolvation/emulsification process, its hydrophilic loops and tails extend into the aqueous continuous phase, creating a dense, hydrated steric barrier around the hydrophobic PLA core.^44^ When two nanoparticles approach due to Brownian motion, the interpenetration of these hydrated gelatin layers generates a significant repulsive osmotic force and a loss of conformational entropy, effectively preventing irreversible aggregation and coalescence, despite the low surface charge.^45^ Consequently, despite exhibiting a moderate positive zeta potential (+16.10 mV), which would indicate borderline stability in a purely charge-stabilized system, the nPLA-G/ClAlPc suspension remained completely stable without particle aggregation or phase separation over 270 days. This confirms that the gelatin chains on the nanoparticle surface provide steric stabilization, forming a hydrated layer that effectively prevents coalescence. Khramtsov et al.^46^ prepared magnetic nanoclusters coated with gelatin and reported that there was no distinct relationship between the zeta potential and its aggregation state. Furthermore, even at high ionic strengths, the zeta potentials withstood aggregation despite being extremely low compared to the well-acknowledged ±30 mV stability threshold, suggesting that steric forces perform a more significant role than electrostatic repulsion. Ultimately, our results showed no significant differences (*p* > 0.05) in size when comparing values at the beginning and end of analyses, confirming that the steric barrier effectively suppresses irreversible aggregation during long-term storage.

The FTIR spectra revealed the characteristic bands of PLA and gelatin in the nPLA-G, confirming the formation of the bionanocomposite. For PLA, we could identify the bands at 1753 cm^–1^ (stretching C=O of the ester group), 1500–1360 cm^–1^ (CH_3_), 1182–1084 cm^–1^ (asymmetric stretching of C–O–C), 1208 cm^–1^ (asymmetric stretching of C–O–C and CH_3_), and 1265 cm^–1^ (deformation of CH and stretching of C–O–C). The three main signals of the functional group amide (CONH) can be found for gelatin: 1633 cm^–1^ (stretch of C=O in amide I with contributions of CN, CCN, and NH), 1533 cm^–1^ (NH scissoring and CN out-of-phase vibration in amide II with contributions of CO and CC), 1444 cm^–1^ (NH and CN vibrations in amide III), and a large peak around 3000–3600 cm^–1^ (−NH and −OH stretching).^47–50^

The FTIR spectra of the nPLA-G/ClAlPc sample maintained the characteristic bands described for the nPLA-G and those referring to the ClAlPc, indicating success in incorporating the PS in the bionanocomposite. For ClAlPc, it was possible to identify 1652 cm^–1^ (C=O), 1600–1300 cm^–1^ (benzene ring skeletal stretching), 1300–1000 cm^–1^ (C–H in-plane bending), 700–800 cm^–1^ (C–H out-of-plane bending vibrations in the phthalocyanine ligand structure), 600–500 cm^–1^ (out-of-plane bending vibrations of the benzene ring), 880–900 cm^–1^ (rocking vibration of the Al-phthalocyanine nucleus), and 500 cm^–1^ (Al–Cl bond).^51,52^

Even though many of the bands in the Raman spectra of PLA and gelatin overlap, we were able to distinguish the compounds in the nPLA-G. PLA’s characteristic bands were 2890, 2946, and 3000 cm^–1^ (CH_2_ stretching vibration), 1765 cm^–1^ (C=O stretching vibration), 1453 and 1300 cm^–1^ (CH_3_ bending vibration), 1127 and 1044 cm^–1^ (C–C stretching vibration), and 873 cm^–1^ (C–COO stretching vibration).^21,53^ The key spectral signatures of gelatin were 1669 cm^–1^ (amide I), 1330 and 1421 cm^–1^ (amide II), 1245 cm^–1^ (amide III), 1450 cm^–1^ (C–H arrangement’s bending vibration), and 2880, 2940, and 2980 cm^–1^ (C–H stretching vibration).^54,55^ It is worth noting that Raman spectroscopy was employed exclusively to confirm the structural biopolymer backbone of nPLA-G. Raman measurements for free ClAlPc and nPLA-G/ClAlPc could not be acquired due to local laser-induced photoreaction/degradation and severe background signal of the photosensitizer under the 1064 nm excitation beam.

PLA’s XRD pattern revealed two distinctive major peaks at 16.8° and 19.2°, indicative of a semicrystalline structure.^56,57^ Gelatin, which lacks a crystalline structure, exhibited taro peaks around 20°.^58^ When the two were combined in the bionanocomposite, the nPLA-G exhibited peaks at the 16.8° and 19.2° positions, and there was no significant difference in the XRD when compared with that of PLA, indicating that the addition of gelatin had no significant effect on the crystallization performance of the bionanocomposites. The XRD of ClAlPc powder showed a peak at 26° that is characteristic of the diffractograms of ClAlPc.^59,60^ The ClAlPc peak in the nPLA-G/ClAlPc indicates that the PS has been successfully loaded into the bionanocomposite.

The purpose of TGA was to determine whether the development of the bionanocomposite resulted in any changes to the thermal profiles, even though the materials will not be applied at high temperatures. The TGA profile of PLA has one step with a maximal weight loss at 366.4 °C (84.85%), with the beginning at 327.4 °C. For the gelatin, degradation occurs in three steps: at 100 °C due to water elimination; at 303.6 °C due to hydrolysis and oxidation; and at 556.5 °C due to decomposition of the polymer backbones into ash.^49,53,56,61^ The nPLA-G exhibited a three-stage degradation profile: at 223.5 °C due to water elimination; at 323.3 °C due to degradation of gelatin and PLA; and at 500–600 °C, indicating decomposition of the remaining polymers into ash.

UV spectra indicate that ClAlPc preserves its spectroscopic characteristics when incorporated into bionanocomposite. The ClAlPc extracted from the nanocarriers perfectly matches the sharp, narrow profile of the free drug standard in DMSO 10%, featuring a well-defined maximum at 676 nm. The absence of a broadened shoulder at shorter wavelengths (H-aggregates) or a red-shifted peak (J-aggregates) indicates that the formulation process successfully prevented self-aggregation, preserving the highly photoactive monomeric state of the photosensitizer within the polymeric core. It is worth noting that preserving ClAlPc in its monomeric form within the bionanocomposite is a key factor for its photoactivity, as self-aggregation typically leads to excited-state quenching and a drastic reduction in singlet oxygen production in aqueous environments. Since monomeric ClAlPc is widely recognized for its high singlet oxygen quantum yield, maintaining this nonaggregated state serves as an indirect spectroscopic indicator suggesting preserved photochemical functionality. Nevertheless, while the present work focused on formulation development, long-term colloidal stability, and primary phototoxic response, direct quantitative tracking of ROS kinetics remains a clear scope limitation of this proof-of-concept study. Consequently, definitive assertions regarding the operative photodynamic mechanism require further mechanistic evaluations. Furthermore, this strong absorption maximum falls strictly within the tissue-optical therapeutic window (650–850 nm), ensuring efficient light penetration for photodynamic activation.^62^

The fluorescence emission spectra of ClAlPc and nPLA-G/ClAlPc indicated maximum emission at 683 nm. The electronic transition of the PS from its ground state to a short-lived excited singlet state is driven by the absorption of energy at a specific wavelength. The subsequent deactivation back to the ground state involves the dissipation of this absorbed energy, which is partially emitted as fluorescence. Therefore, fluorescence is a crucial requirement for a successful PS.^63^ The fluorescence results also indicate that ClAlPc preserves its characteristics when incorporated in the nanoparticles.

The encapsulation efficiency of the proposed system was 90.89 ± 7.55%. Since PLA and ClAlPc are both hydrophobic, encapsulating them in nanoparticles containing PLA is compatible.^64^ Oliveira et al.^23^ produced PLA nanoparticles with an encapsulation efficiency of 0.3 ± 0.8% for zinc phthalocyanine, whereas Ambrosio et al.^24^ achieved an efficiency of 57.8% for ClAlPc in hydroxyapatite microspheres.

The in vitro release studies were performed in two distinct buffers to replicate physiological conditions (pH 7.4) and the environment of endo/lysosomal compartments following cellular internalization (pH 5.0).^65–67^ The analysis of the ClAlPc release profile from nPLA-G/ClAlPc showed a distinct biphasic behavior in both media. At pH 7.4, a burst release of approximately 24% occurred within the first 6 h, followed by a sustained, slow release over 9 days that did not exceed 56%. Conversely, when nPLA-G/ClAlPc was incubated at pH 5.0, 65% of the drug was rapidly released within the first 6 h, exceeding 80% over 9 days. These results indicate that while the overall release trend of ClAlPc remains similar in both media, the photosensitizer release rates are significantly higher at pH 5.0 than at pH 7.4. Phenomenologically, according to literature, this biphasic profile indicates a release mechanism governed by a combination of drug diffusion and matrix erosion. At pH 5.0, the enhanced release rate is strongly favored by the swelling, protonation, and structural relaxation of the hydrophilic gelatin shell under acidic conditions, which expands the polymeric network and accelerates the matrix mass transfer.^20^ Similarly, Khoz et al.^20^ prepared quercetin-loaded gelatin/agarose/Fe_2_O_3_ hybrid nanocarriers and observed that 40% of the drug was released at pH 5.4 after 24 h compared to 25% at pH 7.4, a trend consistent with the behavior of nPLA-G/ClAlPc. Crucially, from a biological standpoint, a clear distinction must be made between the extracellular tumor microenvironment and intracellular compartments. While the extracellular matrix of solid tumors typically exhibits only mild acidity (pH 6.5–7.0), the more pronounced acidic condition of pH 5.0 accurately mimics the endo/lysosomal environment encountered after endocytic cellular uptake.^67^ Therefore, the accelerated release observed at pH 5.0 does not reflect drug accumulation in the extracellular tumor milieu, but rather an engineered intracellular release trigger. This behavior is highly desirable, since at physiological pH (7.4), ClAlPc remains stably entrapped within the nanocarrier matrix, minimizing premature leakage and nonspecific exposure during formulation delivery. Once the nanocarriers are internalized by target cells via endocytosis, the endo/lysosomal acidic pH triggers rapid gelatin matrix swelling and degradation, leading to efficient intracellular liberation of the photosensitizer directly into the cytoplasm, which is ideal for maximizing subsequent photodynamic therapy efficacy.^68,69^ Furthermore, while dynamic serum-induced interactions were not explicitly monitored in real-time in this study, the outer hydrophilic gelatin shell plays a crucial role in biological environments. Literature widely documents that such hydrophilic macromolecular coatings provide a steric barrier that significantly reduces nonspecific protein adsorption and minimizes detrimental protein corona formation or particle aggregation in biological fluids. Additionally, this protective shell helps maintain the physical integrity and structural stability of the nanocarrier during cell culture incubation. Together, these characteristics prevent premature destabilization in physiological media, ensuring that the nanostructure remains intact until reaching the acidic intracellular compartments to exert its photodynamic action.^42,70–72^

After 1 h of incubation, confocal microscopy confirmed the intracellular localization within the cytoplasm for both ClAlPc and nPLA-G/ClAlPc. Furthermore, confocal analysis indicated an enhanced internal fluorescence accumulation when ClAlPc was delivered via nPLA-G/ClAlPc compared to the free photosensitizer. These results align with the known water insolubility of free ClAlPc and highlight the role of polymeric delivery systems in enhancing drug permeation and intracellular retention in melanoma models.^26,66^

To evaluate the overall phototoxic efficacy of the nPLA-G/ClAlPc system, the Trypan blue exclusion assay was strategically utilized as a direct indicator of cell death via cell membrane integrity breakdown. It is worth noting that metabolic colorimetric methods, such as the tetrazolium-based MTT assay, rely heavily on mitochondrial dehydrogenase activity to reduce tetrazolium salts into formazan crystals. However, intracellularly localized photosensitizers like ClAlPc can directly impair mitochondrial function and cellular metabolism immediately upon photoactivation, potentially causing premature alterations in metabolic readout before irreversible cell death occurs.^73^ This dependency can introduce artifacts, leading to misinterpretations or inaccurate over/underestimations of actual cell survival rates. By targeting the terminal outcome of PDT-induced irreversible membrane damage rather than active cellular metabolism, the nonmetabolic Trypan blue assay provided an independent, objective measurement of cell viability under conditions of altered organelle function.

The study of cell viability in SK-MEL-28 cells reveals distinct behaviors between the groups kept in the dark and those exposed to irradiation. In the dark group, cell viability was close to 100% under all experimental conditions. These results show that, in the absence of irradiation, both ClAlPc and nPLA-G/ClAlPc had no significant cytotoxicity to SK-MEL-28 cells at the tested concentrations, indicating that the compounds are biocompatible.

In contrast, in the irradiated group, a pattern clearly dependent on compound type and concentration is observed. As shown in Figure 7b, the irradiated control group (cells exposed to light alone without nanoparticles) and the irradiated nPLA-G group (cells incubated with empty bionanocomposites and exposed to light) both maintained cell viability close to 100%. These results systematically confirm that neither the red LED light dose nor the empty PLA/gelatin bionanocomposite possesses inherent cytotoxicity or photodynamic activity against SK-MEL-28 cells, validating that the observed therapeutic effect is exclusively driven by the photoactivation of encapsulated ClAlPc. Aliakbarzadeh et al.^74^ made gelatin-based platforms for tissue regeneration and showed the biocompatibility of these materials.

The groups treated with ClAlPc and irradiated showed high viability, close to the control, with only a slight reduction to 90% at the highest concentration (1.0 μmol L^–1^). These data suggest that ClAlPc, even under irradiation, does not exert a significant cytotoxic effect under the experimental conditions used. On the other hand, the nPLA-G/ClAlPc under irradiation reveal a concentration-dependent cytotoxic effect. At the lowest concentration (0.35 μmol L^–1^), cell viability was approximately 90%. At the intermediate concentration (0.70 μmol L^–1^), cell viability is reduced to around 80%, with increased experimental variability. At the highest concentration (1.0 μmol L^–1^), a marked reduction in cell viability is observed (*p* < 0.0001), reaching around 3% viability and demonstrating effective, light-triggered phototoxic activity of the nPLA-G/ClAlPc system in SK-MEL-28 cells. These results demonstrate that nPLA-G/ClAlPc exhibits a pronounced photodynamic effect on SK-MEL-28 cells, whereas ClAlPc does not induce significant cytotoxicity, even after irradiation. Additionally, it demonstrates that cell viability depends on the combination of compound type, concentration, and light exposure, reinforcing the potential of the bionanocomposite.

Despite the pronounced phototoxic efficacy observed against SK-MEL-28 cells, nontumorigenic cell models, such as human dermal fibroblasts or keratinocytes, are needed to firmly establish the selective cytotoxicity profile of the platform. While the bionanocomposite demonstrated strong biocompatibility and a complete absence of dark toxicity in the primary evaluation, comprehensive multiassay cytotoxicity profiling (such as metabolic and membrane integrity assays combined) and testing against healthy skin cell lines are necessary for subsequent preclinical phases to precisely define the therapeutic window, safety margins, and true selectivity of the nPLA-G/ClAlPc system.

To contextualize the performance of the developed nPLA-G/ClAlPc bionanocomposite, a comprehensive comparison with other delivery systems reported in the literature was performed (Table 1). This analysis benchmarks critical parameters, such as hydrodynamic size, zeta potential, encapsulation efficiency, release profile, and biological outcomes.

**Table 1:** Comparative Summary of Different Phthalocyanine Delivery Systems from Literature versus the Proposed nPLA-G/ClAlPc Bionanocomposite

Ref	Carrier Type	PS	Size (nm)	Zeta Potential (mV)	EE	Release Behavior	Target Model	Light Dose (J·cm^–2^)	Phototoxicity
Siqueira et al.^11^	Liposomes	ClAlPc	∼110–160	–5 to +2	∼85%	∼80–90% at 24 h (rapid bilayer leakage)	*Leishmania amazonensis*	0–95	4–5 log_10_ reduction in parasite viability
Siqueira et al.^11^	Nanoemulsions	ClAlPc	∼150–250	–10 to −25	∼90%	∼70% at 24 h (oil-to-water partitioning)	Fungal/Bacterial pathogens	10–30	3–4 log_10_ microbial reduction
Cavalcante et al.^10^	Chitosan Nanoparticles	ClAlPc	∼240–285	∼+40	N/A^a^	∼40% at 24 h (sustained matrix diffusion)	*Streptococcus mutans* biofilm	100	5.2 log_10_ reduction in biofilm viability
Paula et al.^26^	Nanoparticles of poly(d,l-lactide-co-glycolide) (PLGA), poly(d,l-lactide) (PLA) and polyethylene glycol-block-poly(d,l-lactide) (PLA–PEG)	ClAlPc	∼115–274	–37 to −59	57%–96%	50–80% within 24 h	Human fibroblasts	10	80% cell death at 1.0–5.0 μmol·L^–1^
Carvalho et al.^22^	Gelatin Nanoparticles	ClAlPc	∼200–300	+34	∼76%	N/A^a^	Macrophage cell line J774A-1	10	85% cell death at 1.0 μmol·L^–1^
Oliveira et al.^23^	PLA Nanoparticles	Zinc Phthalocyanine	∼385	–27	∼80%	∼38% at 24 h (slow PLA erosion)	9L/LacZ gliosarcoma cells	10	80% cell death at 3.0 μg·mL^–1^
Ambrósio et al.^24^	Hydroxyapatite Microspheres	ClAlPc	N/A^a^	N/A^a^	∼57%	N/A^a^	9L/LacZ gliosarcoma cells	10	98% cell death at 250 to 62.5 μg·mL^–1^
Palanikumar et al.^69^	PLGA core with a cross-linked bovine serum albumin (BSA) Nanoparticles	doxorubicin-triphenylphosphonium (Dox-TPP)	∼150–200	–28 to −16	N/A^a^	75% at pH 5.0 vs 10% at pH 6,5 both at 24 h (pH-responsive release)	Human breast cancer MCF-7 cells	N/A^a^	∼30% cell death at pH 7.4 and 90% at pH 6.5 (both at 2 μg·mL^–1^)
Current Work	nPLA-G	ClAlPc	∼809 nm	+16	90.89%	65% at pH 5.0 vs 24% at pH 7.4 both at 6 h (smart pH-triggered)	Human Melanoma (SK-MEL-28)	10	97.0% cell death at 1.0 μmol·L^–1^

a N/A: Not reported/not applicable in the cited study.

The state of the art for DDS applied to photomedicine and cancer covers a wide range of structural options designed to carry various types of substances. Microsized inorganic options, as hydroxyapatite spheres,^24^ function primarily as macroscopic structural depots. On the other hand, liposomes and nanoemulsions^11^ provide mobile delivery but remain vulnerable to thermodynamic leakage and drug crystallization over time. While synthetic polymeric nanoparticles^23,26^ offer controlled sustained release, they typically exhibit faster hydrolytic degradation and lower long-term drug-retention compared to our hybrid design. The developed hybrid nPLA-G bionanocomposite efficiently addresses these performance trade-offs for ClAlPc delivery. By merging the structural integrity of a dense hydrophobic PLA core with the active colloidal stabilization and cell-binding of a gelatin shell, this specific intermediate configuration yields an exceptional encapsulation efficiency of 90.89% and a remarkable shelf life stability of 270 days without any dark toxicity, acting as a highly robust and smart pH-responsive alternative for targeted photodynamic therapy.

## Conclusions

In conclusion, we successfully engineered a PLA/gelatin bionanocomposite formulation with full physicochemical characterization and primary in vitro photodynamic evaluation of a PLA/gelatin bionanocomposite carrying ClAlPc. The nanoparticles were spherically shaped, uniformly sized, and stable over time. The system demonstrated high encapsulation efficiency and pH-sensitive controlled release. The in vitro studies demonstrated that SK-MEL-28 cells responded well to photodynamic treatment when the nPLA-G/ClAlPc was photochemically activated. These findings highlight the potential of the proposed nanocarrier as a platform for ClAlPc delivery, combining advanced polymer engineering with preliminary in vitro evaluations for phototherapy. Ultimately, this study supports further in-depth preclinical investigation to thoroughly quantify ROS generation dynamics, establish the therapeutic window, and clarify the biological selectivity profile of the system.

## Data Availability

The data are available from the corresponding author upon reasonable request.
